# Saying “I Don’t Know”: A Video-Based Study on Physicians’ Claims of No-Knowledge in Assisted Reproductive Technology Consultations

**DOI:** 10.3389/fpsyg.2020.611074

**Published:** 2021-01-12

**Authors:** Julia Menichetti, Jennifer Gerwing, Lidia Borghi, Pål Gulbrandsen, Elena Vegni

**Affiliations:** ^1^Institute of Clinical Medicine, Faculty of Medicine, University of Oslo, Oslo, Norway; ^2^Health Services Research Unit - Akershus University Hospital, Lørenskog, Norway; ^3^Clinical Psychology, Department of Health Sciences, University of Milan, Milan, Italy

**Keywords:** ART consultation, infertility care, lack of knowledge, uncertainty, video-based study

## Abstract

**Introduction:**

The assisted reproductive technology (ART) field deals with consistent and predictable gaps in knowledge. Expressing lack of knowledge with a sentence like “I don’t know” can be challenging for doctors. This study examined physicians’ negative epistemic disclaimer “non lo so” in Italian ART doctor-couple interactions. In particular, it aimed to reveal specific features of “non lo so”: function, topic, temporality, responsibility, and interactional aspects.

**Methods:**

This was a video-based observational study. We used microanalysis of face-to-face dialogue to analyze 20 purposively selected triadic consultations from a corpus of 85. This inductive analysis focused on the function, the content (topic and temporality) and some selected interactional aspects of the “non lo so”, quantifying and capturing the interaction between these qualitative features.

**Results:**

We found 82 doctors’ “non lo so” in the corpus (mean = 4.4; range = 0–15). We discovered three main functions of this expression: propositional (*n* = 73/82), relational (*n* = 6/82), discursive (*n* = 3/82). The most frequent topics raising doctors’ “non lo so” were costs (*n* = 11/82), treatment-related aspects (*n* = 10/82), and timing issues (*n* = 9/82). In more than half of the cases (*n* = 44/82), present issues emerged. The majority (*n* = 70/82) of “non lo so” was framed using the “I,” with doctors’ taking personal responsibility. Patients played a role in these expressions from doctors: Patients initiated more than one third of them, and in one fourth of the cases, patients followed up immediately.

**Conclusion:**

Our findings may be related to characteristics of the specific field of ART. Doctors in this setting must frequently express a direct lack of knowledge to their patients, and when they do, they mean it literally. Patients contribute to such disclosures, and their responses suggest that they find them acceptable, showing that they may expect limitations in their potential to conceive.

## Introduction

In recent decades, medicine has been invited to embrace a complex view of reality and to deal more effectively with uncertainties and limited knowledge (Simpkin and Schwartzstein, 2016). Particularly, doctors and patients expect to discuss treatment options and multiple possibilities: increasingly, there is not just one clear treatment road, rather multiple ones among which to choose (Tinetti et al., 2019). Words like *option*, *risk*, or *decision* now constitute core aspects of clinical practice, all conveying intrinsic uncertainty and a lack of a single, clear, unique direction. Uncertainty in the medical consultation goes along with expanded medical and technological possibilities (Henry, 2006). Via the internet, all kinds and qualities of information become available, rendering the opinion of one professional only a single voice in a sea of voices. On one hand, medicine is forced to move from linearity to complexity, from one medical indication to multiple options; on the other hand, individuals have expanded limitations and possibilities, more information, more influence, fewer boundaries, fewer limitations. Embracing complexity and uncertainty requires a huge shift in mentality, both for doctors and for patients (Henry, 2006; Sturmberg, 2019). In contemporary medicine, doctors must embrace uncertainty and knowledge limitations, rather than preserving the traditional norm of these as negative concepts. New models of practice, such as shared decision-making and a patient-centered consultation approach, encourage doctors to make this shift (Charles et al., 1997).

The challenge of embracing knowledge gaps in the clinical consultation is particularly evident in the field of assisted reproduction technology (ART). In medicine, the ART field is unique: As a medical field born in the 1980’s, it can be considered relatively young. The timing of its emergence means it is more attuned to the complexity of contemporary reality, a characteristic fitting with the deep knowledge gaps practitioners and patients face. Moreover, the consumerism culture seems to find its best medical expression in this field: individuals purchase an expensive medical service (the possibility of procreation), and clinics compete to supply these medical goods (Takhar and Houston, 2019). Doctor-shopping behaviors are frequent (Klitzman, 2017), as is outsourcing and reproductive tourism (Deech, 2003), due to country-specific regulations regarding permitted treatments. ART is a medical possibility that is rarely fully covered by national health insurance schemes, thus patients often pay considerable amounts of money (Kerr, 2013). In addition, the patient is often a couple, two distinct persons with different histories and desires. Patients want and expect more, have higher socio-economic status, higher levels of health literacy, and a larger ability to obtain information (Goisis et al., 2020). Compared to other populations of patients, those seeking ART have more power and less of a disposition to accept failure, uncertainty, and risk. Such patients may not respond favorably to doctors who express a lack of knowledge; as motivated consumers who want clearer answers, patients can go to another doctor. Doctors lose a client, but patients enter a psychologically exhausting doctor-shopping cycle, searching for the doctor who can provide hope (Klitzman, 2017).

In general, the medical field lacks research on what happens in the consultation room, when doctors actually share and manage knowledge gaps and uncertainty while talking to patients (Politi et al., 2007; Han et al., 2019). Indeed, to the best of our knowledge, there are no findings on the topic in the field of ART.

### When the Expert Does Not Know

When doctors say, “I don’t know,” they express recognition of limited scientific, professional, existential, personal, or practical knowledge. If uncertainty is “the subjective consciousness of ignorance” (Han, 2013), saying “I don’t know” represents its most direct and clear communication. In the literature, scholars have studied “I don’t know” expressions primarily from a linguistic standpoint, using ordinary conversations. Linguists have moved beyond this specific expression’s literal signification (not knowing something), disentangling that function from others, such as indexing disagreement, reluctance to cooperate, or desire to close sequences of talk (Tsui, 1991; Beach and Metzger, 1997; Keevallik, 2006, 2011; Weatherall, 2011; Helmer et al., 2016; Lindström et al., 2016). Indeed, speakers can use “I don’t know” even when they actually know, deploying the expression to indicate an epistemic rupture or tension; that is, speakers can use it to hint that they lack certainty about what they have said. This expression has thus been generally conceived as a “negative epistemic disclaimer,” akin to “I don’t remember” or “I don’t understand” (Lindström et al., 2016).

Medical interactions can be conceived as meetings between different epistemologies (i.e., lay and expert knowledge), with asymmetries in knowledge defining power roles in the interaction (Lindström and Karlsson, 2016; Haw et al., 2018). The only study exploring this expression in medical interactions from Sweden, focused on “jag vet inte” expressed by patients, concluding that patients used this expression to claim their epistemic rights and address epistemic tensions and asymmetries in the interaction with their doctors (Lindström and Karlsson, 2016). Thus saying “I don’t know” not only expresses a relationship with knowledge (as lacking or as uncertain) but also shifts or breaks the right to that knowledge, redefining access to power (Lindström and Karlsson, 2016).

Such findings suggest that the expression “I don’t know” may play an important role in the medical encounter, communicating uncertainty and lack of knowledge and organizing epistemic rights and power. By implication, claims of not knowing and uncertainty could reduce or enhance patients’ involvement in the medical encounter and care process. Communicating knowledge gaps and limits can be challenging and against expectations, though more and more necessary as outlined above. It is thus astonishing to observe the lack of studies focusing on doctors’ direct claims of no-knowledge. Empirical studies on doctors’ expressions of uncertainty have instead included a wide selection of uncertainty expressions (Gordon et al., 2000; Medendorp et al., 2018, 2020). While this strategy gives a broader picture of uncertainty, it makes it difficult to disentangle the functions and effects of the most direct expressions in the clinical interaction. As outlined above, saying “I don’t know” does not necessarily mean the speaker lacks knowledge. There is little empirically available evidence regarding how often doctors say “I don’t know” to their patients and what these disclaimers refer to. It is also unclear whether doctors express these disclaimers spontaneously, what role patients play eliciting them, and the immediate effects in the interaction. The peculiarities of the ART field make such expressions particularly salient. Especially in this setting, knowledge gaps may not be the direct responsibility of the doctor but rather a matter of what medical knowledge is available in general or in a given clinic. Thus, there may different degrees of expressed responsibility for the lack of knowledge.

With this study, we examined the physicians’ negative epistemic disclaimer “non lo so” (“I don’t know” in Italian) in ART doctor-couple interactions. We aimed to reveal specific features of “non lo so” (function, topic, temporality, responsibility, interactional aspects) and to answer the following research questions:

i What is the immediate communicative function of the “non lo so”?
ii To what does the “non lo so” refer (in terms of topic and temporality)?
iii What is the interactional surrounding of the different types of “non lo so” and how much does the doctor take responsibility for it, from a literal linguistic perspective?

## Materials and Methods

### Dataset and Sample

The data consist of a subsample of 20 medical interactions with a total length of 15 h from a corpus of 85 collected in eight private and public ART clinics in Italy between 2013 and 2015 (see Leone et al., 2018 for further information regarding the larger research project). The corpus was video recorded and collected with the informed consent of all participants, who gave their consent to use their video for other communication studies. The research project was approved by the Ethical Review Board of the University of Milan and by the Ethical Review Boards of the eight participating ART clinics. Briefly, we selected the subsample analyzed here purposively: First, assuming that the number of people involved can change the doctor’s disposition to express no-knowledge, we aimed to maintain the relational context constant, selecting only triadic visits. Second, we did not know the effect familiarity with the patients would have on whether doctors would express lack of knowledge; thus, we selected an initial and a follow up from the same doctors (although not necessarily the same patients). Applying these criteria to the 25 physicians (females *n* = 15, 64%; mean years of experience = 17.8), ten (females *n* = 7, 70%; mean years of experience = 16.6) were found to have both a first and a follow-up triadic visit. The subsample of data was verbatim transcribed from videos, using selected Jefferson notations (i.e., pauses, overlaps, cut-offs, continued turn, prolonged vowel/consonant, unclear word, notes and descriptions) (Jefferson, 2004). The extracts reported have a literal word-by-word English translation, an idiomatic translation is supplied in case the word-by-word translation obscures the meaning of the Italian.

### Method of Analysis

We used microanalysis of face-to-face dialogue (Bavelas et al., 2016) to analyze the video recordings. While this methodological approach emerged from experimental social psychology (Bavelas et al., 1986), it has theoretical roots in social constructionism (Berger and Luckmann, 1966), symbolic interactionism (Blumer, 1969; Caglar and Fuson, 2015), and pragmatics, in the sense that it is concerned with how interlocutors’ make meaning from each other during ordinary language use (Levinson, 1983). Most broadly, the analysts’ interpretation of behaviors in interaction is guided by both the collaborative model of communication (Clark, 1996) and the integrated message model (Bavelas and Chovil, 2000).

Microanalysis of face-to-face dialogue is suited to investigating the processes and content of communication. The goal of a comprehensive, inductive microanalysis is to find all manifestations of the phenomenon of interest and to characterize them along relevant dimensions. The microanalytic lens focuses analysts on what they can observe directly from the video (i.e., interlocutors’ words, how they say them, and what they do with their body at the same time). In this way, analysts concern themselves with the observable *what, when*, and *how* of the behaviors of interest rather than the underlying *why’s*. Thus, for this study, the focus was stringently on when and how physician’s uttered “non lo so”, rather than on their actual state of knowledge, much less their motivations or intentions. The microanalytic lens further assumes that behaviors are polysemous, with meanings that can only be gleaned from context. In this case, even though the form of “non lo so” was more or less fixed (see details below), inferring what physicians conveyed when uttering those words depended on attending to their immediate interactive context, including the topic under discussion and what happened immediately previously and after.

The process of microanalysis begins by using the phenomenon of interest as a concrete *entry point* into the videotaped interaction (i.e., doctor utterances of “non lo so”). Through a process of collecting and constructing a definition of the salient features of that phenomenon, analysts eventually collect all instances. Then, through careful comparison of the instances and their immediate sequential context, the analyst can decide how best to characterize them, highlighting differences that could be most relevant for the overall purpose of analysis (in this case, to inform clinical practice). While an *a priori* categorization can be set (e.g., the topic to which the “non lo so” refers, which was informed by the taxonomy of Han et al., 2011, see details below), some emerge as important only during the analytical process. For example, here the *function* became relevant when the main analyst realized that not all “non lo so”s seemed to function to convey a lack of epistemic knowledge. Nevertheless, in the description of analysis and results, we do not distinguish between features and categories that were planned and those that emerged during analysis.

### Data Analysis

Transcripts of videos along with videos were scrutinized by one researcher (JM) for extracts where the doctor expressed a “non lo so”/“non lo sappiamo”/“non si sa” (“I don’t know”, “we don’t know,” “it is not known”). Inclusion of slightly different versions of the formulations or of doubtful cases was discussed with a small group of five researchers with experience in video-analysis of medical consultations. In general, expressions changing the meaning of the claim were excluded (e.g., “we cannot know,” “how can I know?,” “it is impossible to know this in advance”), while expressions with words in a different order or reduced variants were included (e.g., “non so,” meaning the first person “I do not know” without the object it/“lo” which is usually needed in Italian). Such variants were so close to the original formulations that their inclusion was straightforward, considering that in Italian, the subject of a sentence is expressed both in the subject pronoun and/or, most often, in the verb conjugation. When the final set of extracts was defined, the same researcher analyzed the linguistic features and contents of the included expressions. Another researcher (LB) analyzed a random sample of 20% of the included “non lo so” independently, and disagreements were solved by discussing them and were used to refine the analysis.

To contextualize the features of “non lo so,” we present an example of a doctor who is expressing his lack of further treatment options to a couple while they report a possibility of treatment in another clinic.

**Table:** 

106	Doctor (D)	[ma con il collega che cosa] ha consigliato di [stimolazione] *But the colleague what did he suggest as stimulation*
107	Female patient (FP)	[aveva proposto] un altro protocollo *He suggested another protocol*
108	D	eh (.) ok *eh ok*
109	FP	eh [noi ve lo facciamo vedere se possiamo perché tanto noi] *eh we can show it to you if we can because in any case we*
110	Male patient (MP)	[(no ce l’ho io, no ce l’ho io)] *no I have it, no I have it*
111	D	oh a me è tutto arricchimento eh. **Io non so, non so** più [che fare] (ride) *oh for me it is all enriching eh. **I don’t know, I don’t know** what to do anymore (laughs)*
112	MP	[ce l’ho io] *I have it*
113	FP	grazie (sorridendo) [consolante] *thank you (smiling) [comforting]*
114	D	[nel senso] cioè quello che noi si pensava che potessero essere le cose che funzionavano di più le abbiamo provate quindi- *I mean we tried what we thought it would have worked most so*

The extract foreshadows and illustrates four key features of “non lo so” analyzed in this study: it touches on aspects of *content* (what is not known), *temporality* (whether the “I don’t know refers to the past, present, or future), *responsibility*, and *function* (what it is doing in the interaction at that moment). The patients play a role, sometimes initiating and always responding; analysis took into account these *interactional* features as well.

The *topical content* of each instance of “non lo so” were analyzed by combining a deductive and an inductive approach. Contents related to the “non lo so” were extracted inductively (from the object complement, if present, or from the related question or close topic) and organized in bottom-up categories, which were at the end grouped in macro-categories based on a taxonomy of substantive issues of medical uncertainty (Han et al., 2011). According to this taxonomy, three substantive categories feature medical uncertainty: (i) scientific, (ii) practical, (iii) personal (Han et al., 2011). While some relational contents of the “non lo so” also emerged from the data (e.g., a doctor responding “non lo so” to a patient question about why she refer to the female patient with the informal pronoun “you” and to the male patient with the formal third person), these were ultimately collapsed into the personal category. In line 111 of the example above, the doctor expresses lack of knowledge about *scientific/medical content*, specifically available treatment options.

The *temporality* (past, present, future) of the “non lo so” was extracted based on the grammatical indicators used in the sentence (e.g., verb, temporal adverbs). In the example, the doctor referred to present matters (e.g., options, treatments, possibilities), saying he does not know what to do anymore.

The *function* of the “non lo so” was positioned at an illocutionary level and anchored on selected linguistic descriptors of the “non lo so”: (1) with vs. without object complement; (2) the sequential position, (3) in responsive vs. first position turns. The linguistic descriptors were complemented by the analyst’s understanding of the interaction dynamics as preceding and following turns were considered. While initially the categories of functions drew from previous studies of this expression in other fields (Tsui, 1991; Beach and Metzger, 1997; Keevallik, 2006, 2011; Weatherall, 2011; Helmer et al., 2016; Lindström et al., 2016), in keeping with the inductive nature of the analysis, new categories emerged from the data. The doctor in the above example uses the expression literally, at the propositional level to claim a lack of knowledge. Patients may reveal access to more knowledge than doctors have, as they often are in contact with multiple ART clinics.

In this setting, knowledge gaps may easily not be the direct *responsibility* of the doctor but rather a matter of what medical knowledge is available in general or in a given clinic. Here, the doctor uses the “I” construction, expressing his own responsibility for the lack of knowledge.

Finally, key linguistic and *interactional* aspects were also selected to describe the interactional surrounding of the “non lo so.” Analysis focused on the pragmatic nature of the turn, identifying whether the doctor’s expression was in response to a query or statement from the patient and which participant raised the topic. Capturing the sequential context required including more than the immediate utterances before and after the “non lo so,” as sometimes doctors’ multiple turns when responding to a patient’s question combined with patients’ encouragement to continue constituted responsive turns. Another interactional aspect was the degree of responsibility the doctor claimed, when expressing lack of knowledge to the patient. Here, analysis focused on the pronoun the doctor used, which was either I (an explicit, personal disclaim) or we/impersonal pronoun (a less explicit, more impersonal disclaim). Finally, if and how the patient followed up after the doctor said “non lo so” provided an indication of acceptability (e.g., the patient might follow the immediate topic of the “non lo so”). By examining these interactional surroundings, analysis can reveal the immediate result when doctors reveal epistemic holes directly. In this excerpt, the male patient responds (in line 112) without surprise and with a smile, repeating “I have it”, referring to the suggested protocol. The patients here do not comment specifically on the doctor’s lack of knowledge.

Table 1 summarizes the dimensions of “non lo so”, along with a brief definition and an extract.

**TABLE 1:** Key features of “non lo so” considered in the analysis, with definition and decontextualized examples.

Key feature	Brief definition	Example
***Topic***	***The topic about which the doctor is stating a lack of knowledge***	
*Scientific/Medical*	causal explanations, treatment recommendations, prognosis, examinations, and other health issues	(1) This is a procedure that we usually follow when the sperm liquid is not good, so **we don’t know** why an embryo did not develop from the 10 oocytes that were fecundated (…)
*Practical*	expected quality of care, the structures of care, and the procedures required to access care	(2) But we don’t work in that way, I mean, we treat all the patients in the same way, **I also didn’t know** you were covered by the national health insurance system (…)
*Personal/relational*	From patients’ point of view: the effects of illness or treatment on their personal experiences and goals in life From doctor’s point of view: personal disclosures of limited possibilities of knowledge and action From doctor’s and patients’ point of view: disclosures of limited knowledge about aspects concerning their actual relationship	(3) if to wait or to decide to go abroad: **I do not know** what to suggest to you, surely if you have in mind to go abroad because it’s quicker…
***Temporality***	***The time to which the doctor is referring***	
*Past*	Something that preceded the consultation	See (2) above
*Present*	Something that is occurring during the consultation (e.g., …)	See (1)(3)
*Future*	Something that can happen in the future	(4) **I don’t know** who will meet you the next time (…)
***Function***	***What the “non lo so” is doing in the interaction at that moment***	
*Propositional*	Conveying negative epistemic stance (lack of knowledge, aleatory uncertainty, obtaining information from the patient)	See (1)(2)(4)
*Discursive*	Managing the conversation (marking turn or topic exchange, maintaining the turn, hesitation)	(5) D I give you back the papers, because you understand coming here for a stimulation is one thing FP mh D coming for doing everything MP (unint) FP (unint) D **I don’t know** (.) I will get the information, think about this FP (unint) MP yes (.) (unint)
*Relational*	Managing preferences about roles and positions in the relationship (including the preference of not having a position regarding a certain instance)	See (3)
***Responsibility***	***How the “non lo so” statement places responsibility for the lack of knowledge***	
*Personal*	The doctor uses “I”	See (2)(3)(5)
*Generic*	The doctor uses “we” or the impersonal pronoun	See (1)(6)
***Interactional aspects***	***What precedes and follows the “non lo so”***	
*Who raises topic*	Patient initiates the topic (i.e., the “non lo so” is in the responsive turn) vs. the doctor raises	(6) FP I produce 4 oocytes D but **we don’t know** how many do you produce when you are under stimulation (…)
*Patient follow up*	Patient continues/expands/follows up the topic of the “non lo so” in the next turn vs. not	Next turn of (3) FP I don’t know what to do either, I have a refusal inside me

These aspects were analyzed in Excel and reported by using descriptive statistics (frequency; average; percentage). We selected one extract from one consultation, that we reported in detail, to highlight key aspects emerged from the descriptive analysis.

## Results

Overall, 82 doctors’ “non lo so” were found in the 20 analyzed consultations. There was a median of 2.5 no-knowledge claims per visit (range = 0–15). The majority of the no-knowledge claims was in the first visits (*n* = 50; 61%, with a median of 3, range 2–15), while follow up consultations contained 32 “non lo so” (32%; median = 2, range 0–12). Regarding physicians’ characteristics, the seven female physicians expressed a median of 4 “non lo so” per visit (range 2–15), while the three male physicians expressed a median of 1.5 “non lo so” per visit (range 0–3). When dividing for their years of professional experience, the five physicians with less than 15 years of experience expressed the same median of 2.5 “non lo so” per visit (range 1–15) as those with more than 15 years of experience (range 0–12).

### The Doctors’ “Non lo so” Functions: Propositional, Discursive, Relational

The 82 doctors’ claims of no-knowledge covered three main mutually exclusive functions: propositional, discursive, relational. As will be shown in the following, not all claims had a prototypical function of truly conveying negative epistemic stances like lack of knowledge (“propositional”), but some served discursive functions, managing turns (“discursive”), while others served a relational function, managing roles and positions in the interaction rather than expressing epistemic stances.

The majority of the “non lo so” expressions (*n* = 73/82) had a true propositional function. These were distributed in the following ways. First, doctors primarily used propositional “non lo so”s to convey an outright lack of knowledge (*n* = 49/73), for example, when they were unable to answer patients’ requests for information (either directly or in anticipation of informational needs) or when they communicated areas of ignorance to justify past, present or future actions. Second, doctors used them to express uncertainty about information-containing utterances, terminology, or on-going behaviors (*n* = 17/73). Finally, doctors used them to obtain information from patients (*n* = 7; 10%), expressing a lack of knowledge about something the patient might know and be able to contribute to the discussion.

A few “non lo so” expressions (*n* = 6/82) had a relational function, meaning that they were used by doctors to manage preferences about roles or positions in the interaction, including the preference of not having a position (directly asked or expected to be asked).

A minority of the “non lo so” (*n* = 3/82) functioned as discursive markers. In one case, the doctor used the expression to close the patient’s turn, and twice, a doctor used it to hesitate, allowing the doctor to time to reflect and plan.

### The Doctors’ “Non lo so” Contents: Scientific/Medical, Practical, Personal-Relational Topics and Temporality

Doctors primarily referred to practical topics when saying, “non lo so” (*n* = 40/82), followed by scientific/medical (*n* = 29/82), and personal-relational topics (*n* = 13/82). In particular, the specific categories of topics most frequently raising the “non lo so” were costs (*n* = 11/82), treatment-related aspects (*n* = 10/82), and timing issues (*n* = 9/82). Table 2 provides a description of the type and frequency of doctors’ “non lo so” topics and specific categories.

**TABLE 2:** Type and frequency of doctors’ “non lo so” (*n* = 82).

Main topics and categories	n
***Practical topics***	***40***
costs	11
timing	9
coordination	8
bureaucracy	7
computer	3
location	1
patient attrition/flow	1
***Scientific/Medical topics***	***29***
treatment	10
causes	7
examinations	5
prognosis	5
other health issues	2
***Personal-relational topics***	***13***
patient choice	4
relational aspects	3
living place	2
self-disclosure	2
patient experience	1
doctor choice	1

Regarding the temporality of the doctors’ “non lo so,” most referred to present issues (*n* = 44/82), followed by past (25/82) and future (13/82). Combining these frequencies with those from the topic analysis revealed that the majority of the “non lo so” about scientific/medical topics referred to past issues (*n* = 14/29), while for the other two topics the temporal reference was mostly to the present (*n* = 23/40 for the practical and *n* = 10/13 for the personal-relational topics). Table 3 presents the frequencies of the “non lo so” temporality by the three topics.

**TABLE 3:** Frequency of “non lo so” temporality by topics.

	Temporality		
	Past	Present	Future
Scientific/Medical topics	14	11	4
Practical topics	9	23	8
Personal-relational topics	2	10	1
	25	44	13

### Functions of the “Non lo so” for the Different Topics

When connecting the main functions with the topics of the “non lo so,” we observed that doctors used all scientific/medical and most of the practical (*n* = 36/40) topic-related “non lo so” to literally convey a negative epistemic disclaimer, while personal-relational topics had a greater variation in how doctors used them. Table 4 shows the frequency of uses by the different topics.

**TABLE 4:** Frequency of functions by the different “non lo so” topics.

Function	Topic			Total
	Scientific/Medical (n = 29)	Practical (n = 40)	Personal-relational (n = 13)	
Propositional	29	36	8	73
Discursive	0	2	1	3
Relational	0	2	4	6

### The Interactional Surrounding of the Different “Non lo so” Types

Finally, we explored the interactional surrounding of “non lo so”; in particular, whether the doctors were responding to patients when they said it, how the patients followed up, and how much responsibility the doctor took for the lack of knowledge from a linguistic standpoint (as indicated by pronoun use). Table 5 presents the frequencies for these interactional features.

**TABLE 5:** Frequency of interactional aspects of the “non lo so” (*n* = 82).

Interactional aspects		
“Non lo so” raised by the patient (responsive turn) vs. the doctor (first turn)	“I” vs. “we/impersonal” pronoun (personal vs. deferred responsibility)	“Non lo so” followed up by the patient vs. not
32 vs. 50	70 vs. 12	21 vs. 61

Overall, the “non lo so” were usually raised by doctors (*n* = 50/82) rather than being responsive to something initiated by patients (*n* = 32/82). Almost all of the 32 “non lo so” initiated by the patients had a propositional function (*n* = 30/32). Patients were more likely to open scientific/medical topics raising doctors’ “non lo so” expressions (*n* = 14/29) than practical topics (*n* = 15/40) and personal-relational (*n* = 3/13). This distribution of frequencies was more or less the same when focusing specifically on propositional “non lo so.”

In general, patients did not follow up the “non lo so” in the next turn (*n* = 61/82), and especially not when the “non lo so” was about personal-relational issues (*n* = 11/13). Patients were slightly more likely to follow up the “non lo so” and explore it in the next turn when the topic of the “non lo so” was about scientific/medical issues (*n* = 8/29). Overall, “non lo so” expressions explored by the patients in the next turn had in most of the cases (*n* = 18/21) a propositional function.

Finally, the majority (*n* = 70/82) of the “non lo so” were framed using the “I” pronoun, thus indicating doctors taking a personal responsibility for the lack of knowledge from a literal linguistic perspective. This was the case of all the “non lo so” about personal-relational topics and of most (*n* = 36/40) of the practical ones. In some (*n* = 8/29) of the “non lo so” about scientific/medical topics, the doctor framed the expression deferring responsibility to others using the “we” or impersonal pronoun. All the “non lo so” framed using the “we” or an impersonal pronoun had a propositional function, while all non-propositional functions were framed using the first person pronoun.

### Zooming Into One Consultation

In this section, we report in detail an excerpt from one consultation. In it, the doctor expressed “non lo so” several times, and the excerpt focuses on one that illustrates the dialogic context surrounding this particular “non lo so”, which has a propositional function.

In this consultation, a couple with a diagnosis of infertility asked the gynecologist’s opinion about the possibility of performing a second heterologous fertilization in Italy. While it was the first time that this doctor and these patients met, the couple was not new to the ART field. They previously attempted to conceive with a heterologous fertilization with ovum donation. This attempt was through a different clinic, and the treatment failed. During the consultation with this gynecologist, the couple complained about the lack of information about the treatment failure from the other clinic, and they asked for explanations. Despite not knowing the details of what actually happened at the former clinic, the doctor explained why- in her view- the treatment failed. Extended talk about the medical and practical knowledge limitations about ART unfolded, both about the failed treatment and about the decision to take regarding if, when, and where to undergo a second heterologous fertilization. This extract of a “non lo so” from the end of the visit, seeing the doctor coming back to the desk after having printed some papers, exemplifies how the patients direct the conversation toward making the uncertainty and lack of knowledge more explicit:

**Table:** 

Extract 1 (First visit, female doctor, second level treatment; 0:57:36)		

518	D	(enters in the camera and sits at the desk) quindi fondamentalmente io vi metto in questa nostra lista (.) così però non (Word) avremo informazioni speriamo a settembre o fine luglio (.) pero quali informazioni purtro:ppo = *so basically I put you in our waiting list (.) so but not (Word) we will have the information hopefully in September or end of July (.) but which information unfo:rtunately* =
519	MP	= non lo sapete = *you don’t know*
520	D	**non lo sappiamo** (.) non-possiamo dire con certezza che nel giro di 3 mesi siamo in grado di fare (.) quindi sulla base di questo valutate ***we don’t know*** *(.) we cannot say for sure that in three months we are able to do (.) so evaluate based on this*
521	MP	quindi aspettiamo settembre cosa fa cosa = *so we wait September what does what* =
522	FP	= noi ci mettiamo in questa lista d’atte:sa (Word) [se decidiamo] =*we enlist in this wa:iting list (Word) [if we decide]*

In this case, the “non lo so” expresses a real (propositional) lack of knowledge about a practical topic: bureaucracy, and it is related to a future issue as revealed by the time references in lines 518 and 520. It is one of the few practical “non lo so” framed using the “we” (mirroring the patient’s formulation with the second-person plural pronoun “you” in line 519), which is elicited (line 519) and also followed up by both patients, who rephrase and ask the doctor to specify the implications of the “we don’t know” (line 521 and 522).

Therefore, this extract highlights some of the key points from the previous sections: (1) Patients can be open to explicit expressions of lack of knowledge (in this case, by anticipating them and even completing the doctor’s sentence), (2) Doctors can express varying degrees of uncertainty with “non lo so”, and patients can guide doctors in specifying the degrees of acceptable uncertainty (see 521–522 and the clarification in 520 following the “we don’t know”), (3) In the ART setting, both doctors and patients influence doctors’ expressions of not knowing something, doctors can do so spontaneously or responsively, elicited by patients, and (4) The decision-making process can lean on significant epistemic holes (see 520–522) and does not necessarily depend on notions of certainty expressed during the interaction.

## Discussion

This is the first study analyzing doctors’ negative epistemic disclaimer “I don’t know” in medical consultations. We described distinctive features that accompany the “I don’t know” in the ART setting: content (topic, temporality), function, interactional surrounding.

The first finding of this study is that ART doctors frequently say “I don’t know” to their patients. We detected 82 doctors’ “I don’t know” in 20 consultations, that is, a median of approximately three per visit, with one doctor expressing 15 such expressions in the same consultation. This finding was unexpected, given the literature on the issue. Opinion papers on uncertainty in the medical consultation argue that doctors do not disclose lack of knowledge or uncertainty (Henry, 2006; Lian and Robson, 2019). Indeed, the doctors’ role is to diagnose, evaluate, or treat a patient’s condition; patients typically go to the doctor precisely to seek an expert view on how to get well. As Pilnick and Dingwall (2011: 1374) have argued, “asymmetry lies at the heart of the medical enterprise: it is, in short, founded in what doctors are there for”. As mentioned in the introduction, Lindström and Karlsson (2016) conducted the only empirical study specifically focused on this expression in medical consultations during which patients were seeking relief from rheumatism and fibromyalgia. These authors focused on patients’ “I don’t know”, identifying 29 such utterances in 35 consultations. Empirical studies on doctors’ expressions of uncertainty (not limited to “I don’t know”) in the medical consultation report rates slightly lower than our findings. Gordon et al. (2000) found that doctors made 475 direct verbal expressions of uncertainty in 154 primary care visits, with a mean of 2 per visit. A recent study (Medendorp et al., 2020) on 29 simulated genetic counseling consultations focused on expressions of uncertainty, and these authors found 1207 such utterances in counselors, with 77% of them framed directly (including some “I don’t know” expressions). The pure, formal expression “I don’t know” could be seen as the tip of the iceberg of expressing uncertainty, suggesting that ART consultations may be a particularly rich source for studying such expressions. As the same Gordon et al. (2000) revealed, physicians express more uncertainty to patients with more education, greater desire for information, and more questions, precisely the characteristics of patients seeking ART. Our findings suggest specific training needs for ART doctors, namely how to disclose lack of knowledge and uncertainty. Our findings also demonstrate the potential of the field of ART to reveal current practices of disclosing uncertainty to patients that can be used as natural strategies in other fields. It also provides empirical ground for showing that doctors seem prone to embrace and communicate their lack of knowledge and uncertainty directly.

We found that almost all the ART doctors’ expressions of “I don’t know” conveyed uncertainty or lack of knowledge (i.e., with a prototypical or propositional function). This finding is not consistent with the linguistic literature on the use of “I don’t know” in ordinary (non-clinical) conversations. Indeed, the function of truly displaying lack of knowledge was found to occur in only 7.6% of the 210 instances when speakers expressed “I don’t know” in daily interactions (Helmer et al., 2016). Linguists have concluded that this expression functions much like a discourse marker, serving as an interaction-organizing resource rather than conveying literal meaning (Lindström and Karlsson, 2016). Our contrasting findings highlight how the specific circumstances of the medical interaction influence such expressions, with the particularities of ART consultations providing potentially fertile ground and rationale for this expression. In particular, medical and practical topics, specifically treatments, timing, and costs provided concrete reasons driving the need to communicate lack of knowledge. Our findings around these topics provide concrete indications for ART doctors, pointing to which areas of their work may require the need to express uncertainty to patients, thus helping ART doctors to be prepared to such disclosures.

The findings of this study also highlighted the relational aspects (both in the function and topic) in direct expressions of lack of knowledge. The relational dimension has rarely been mentioned, neither in the literature on medical uncertainty more broadly (Han et al., 2011; Medendorp et al., 2018, 2020) nor in the linguistic literature focused on medical interactions (Lindström and Karlsson, 2016). In Han’s taxonomy of medical uncertainty, the relational dimension notably missing (Han et al., 2011), perhaps due to the way the taxonomy was developed: it was based on existing literature and not on empirical studies observing real medical interactions. Medendorp et al. (2018) used the same taxonomy as the basis for analyzing clinical consultations, without opening the analysis to include novel, emergent phenomena. Lindström and Karlsson (2016) was an inductive analysis of medical interactions, but they used specific lenses (knowledge asymmetries) in the analysis of patients’ use of “I don’t know.” While our findings do not provide indications about the exact and in-depth reasons why doctors express a relational-type of lack of knowledge, we speculate that the reasons may be multiple, ranging from reducing the relational distance from patients to shying away from a difficult conversation. In general, we suggest including relational aspects in studies from linguistics and medicine that focus on expressions of lack of knowledge and uncertainty. Further studies should include non-verbal communication and/or explore doctors’ views on the use of relational types of “I don’t know” to disentangle the reasons behind their use.

Even with a direct, clear, explicit expression such as “I don’t know”, different degrees of uncertainty can be expressed. This was particularly evident by our analysis of the function and topic of the “non lo so”s in our material. Indeed, we found propositional functions ranging from communicating areas of ignorance and epistemic holes to expressing doubt or giving epistemic legitimacy to the patient, using the “I don’t know” to obtain information that was lacking up to that point. The topic indirectly revealed different possible degrees of uncertainty too, ranging from intrinsic and hard-to-reduce knowledge topics (like the scientific/medical ones) to more easy-to-reduce knowledge topics like the practical ones (e.g., the case of costs). Further studies might explore the role of topics and functions in revealing degrees of uncertainty that could be expressed by a same utterance. Interestingly, “I don’t know” about potentially hard-to-reduce topics like the medical ones were quite frequently framed using the first-person pronoun, thus indicating doctors taking a personal responsibility for it. This may support the first finding here discussed: ART doctors seem to be open to face, communicate, and take responsibility for a lack of knowledge, even in cases where the responsibility relies on other people, institutions, or forces.

Finally, our findings revealed some interactional features of the “I don’t know.” More than one third of the “I don’t know” was expressed after a patient request or elicitation, especially when the expression referred to medical topics (close to 50%). In the linguistic literature, the occurrence of this expression has been related to responses to questions, which is considered to be the most frequent sequential environment of an “I don’t know” occurrence (Tsui, 1991; Beach and Metzger, 1997; Lindström et al., 2016). This may indicate that both patients and doctors are willing to discuss knowledge holes, thus revealing the ART consultation as a person-centered place, where both parties have the possibility of sharing and co-constructing care. Such finding exemplifies “patient-centered” aspects of the ART consultation that have not been grasped in previous studies, where the ART consultation was rated as very much “disease-centered” if looking at the topics discussed (Leone et al., 2018).

Finally, we found that patients followed up on only one fourth of the “I don’t know” expressions. This finding could indicate that the patient “accepts” the “I don’t know”, which may ultimately serve to close the topic and open the door to moving on to other relevant issues. This finding could be related to the very specific field of ART: patients are aware there is uncertainty in the treatment they are undergoing and in the prognosis, and they seem to search for limitations in the natural, human possibilities to conceive, rather than exhausting cycles of hope.

## Limitations

This study has some limitations. First, the analysis was focused on verbal communication, and while we considered non-verbal aspects in our interpretation of the speech, we did not include non-verbal aspects specifically in the analysis. This may have limited findings, as non-verbal aspects (e.g., gaze direction, facial displays) can be particularly relevant in explaining and characterizing uncertainty and lack of knowledge expressions. Further studies should include and focus on non-verbal aspects more directly. Second, we included any type of “I don’t know”, without, for example, considering in advance linguistic differences between not knowing whether (believed/uncertain) and not knowing at all (unknown) (Zuczkowski et al., 2017). Third, we analyzed a “fixed” expression, without including other expressions that may have conveyed the same meaning. While this decision may have obstructed any investigation of the different ways physicians express lack of knowledge, it afforded the opportunity for us to reveal the various functional and interactional nuances of a same expression with increased certainty regarding interpretation. Fourth, we extracted the interactional function, but we did not have data about the views of doctors on the reasons why they said “I don’t know” and their intrinsic motivations and intentions in saying it.

## Conclusion

Findings of this study reveal that doctors’ “I don’t know” expressions are frequent; they are mostly used with a propositional function, about present issues and about treatment, costs, and timing; they are framed with doctors’ taking a personal responsibility, they are opened both by patients and doctors, and they are immediately followed up by patients in one quarter of the cases. While not common, relational aspects emerged as relevant topics and functions characterizing the “I don’t know”. Findings provide indications to (ART) doctors about the need to disclose lack of knowledge to patients, about what, and about the openness of the patients and positive patients’ reactions to it.

## Data Availability Statement

The raw data supporting the conclusions of this article will be made available by the authors, without undue reservation.

## Ethics Statement

The studies involving human participants were reviewed and approved by Ethical Review Board of the University of Milan. The patients/participants provided their written informed consent to participate in this study.

## Author Contributions

JM, EV, JG, and PG conceived and designed the study. EV collected and provided the data. JM and LB analyzed the data. JG supervised the data analysis process. JM and JG wrote the manuscript with the input from all authors.

## Conflict of Interest

The authors declare that the research was conducted in the absence of any commercial or financial relationships that could be construed as a potential conflict of interest.
